# The Effects of Seed Ingestion by Livestock, Dung Fertilization, Trampling, Grass Competition and Fire on Seedling Establishment of Two Woody Plant Species

**DOI:** 10.1371/journal.pone.0117788

**Published:** 2015-02-19

**Authors:** Julius Tjelele, David Ward, Luthando Dziba

**Affiliations:** 1 Agricultural Research Council, Animal Production Institute, Irene, 0062, South Africa; 2 School of Life Sciences, College of Agriculture, Engineering and Science, University of KwaZulu-Natal, Scottsville 3209, South Africa; 3 CSIR: Natural Resources and the Environment, Pretoria 0001, South Africa; Henan Agricultural Univerisity, CHINA

## Abstract

The increasing rate of woody plant encroachment in grasslands or savannas remains a challenge to livestock farmers. The causes and control measures of woody plant encroachment are of common interest, especially where it negatively affects the objectives of an agricultural enterprise. The objectives of this study were to determine the effects of gut passage (goats, cattle), dung (nutrients), fire, grass competition and trampling on establishment of *A. nilotica* and *D. cinerea* seedlings. Germination trials were subjected to the following treatments: 1) seed passage through the gut of cattle and goats and unpassed/ untreated seeds (i.e. not ingested), 2) dung and control (no dung), 3) grass and control (mowed grass), 4) fire and control (no fire), 5) trampling and control (no trampling). The interaction of animal species, grass and fire had an effect on seedling recruitment (*P* < 0.0052). Seeds retrieved from goats and planted with no grass and with fire (6.81% ± 0.33) had a significant effect on seedling recruitment than seeds retrieved from goats and planted with grass and no fire (2.98% ± 0.33). Significantly more *D. cinerea* and *A. nilotica* seeds germinated following seed ingestion by goats (3.59% ± 0.16) than cattle (1.93% ± 0.09) and control or untreated seeds (1.69% ± 0.11). Less dense grass cover, which resulted in reduced grass competition with tree seedlings for light, space and water, and improved seed scarification due to gut passage were vital for emergence and recruitment of *Acacia* seedlings. These results will contribute considerably to the understanding of the recruitment phase of woody plant encroachment.

## Introduction

An increase in woody vegetation density [1, 2] known as woody plant encroachment, has been widely reported in southern Africa [3, 4]. The increasing rate of woody plant encroachment in grasslands and savannas is a challenge to livestock farmers [5, 6]. An increase in woody plant density suppresses the productivity of herbaceous plant species [1] and eventually reduces rangeland carrying capacity [1, 7]. The reduction in carrying capacity is of great concern because African savannas have a rapidly growing human population [1]. Despite the common and widespread occurrence of woody plant encroachment [8], its dynamics are not entirely understood, particularly the mechanisms controlling woody plant encroachment and dominance [9, 10]. There is considerable interest in understanding woody plant encroachment [1] in order to improve livestock production.

There is agreement that rainfall, soil, nutrients, herbivory and fire are key variables affecting tree-grass ratios [11, 3]. However, little attention has been paid to the influence of endozoochory (i.e. seeds ingested and subsequently dispersed by animals) on woody plant encroachment [12]. Grazing herbivores reduce the ability of grasses to compete with tree seedlings for resources through grazing [13]. Conversely, herbivores disperse viable woody plant seeds [14], and may increase seedling emergence, seedling establishment and recruitment of woody plant species through gut passage and dung fertilization [15]. Endozoochory, seedling emergence, seedling establishment and recruitment are crucial processes in plant population dynamics because they usually influence the distribution and abundance of plant species [16, 17]. The appearance of a radicle marks the end of seed germination (the emergence and development from the seed embryo) and the beginning of seedling establishment, a period that ends when the seedling has exhausted the food reserves stored in the seed [18, 19]. Seedling recruitment refers to the process by which new individuals/seedlings establish a new population or are added to an existing population [20, 21].

Pods of certain woody plant species form an important part of the diet of goats, and to a certain extent sheep and cattle, and help satisfy their nutritional requirements during the dry season [22, 23]. *Acacia* and *Dichrostachys* seeds have hard seed coats, which enable some of the ingested seeds to be passed out unharmed in the faeces [24]. Herbivores with large body size such as cattle have longer retention time than goats [25]. A positive correlation between germination rates and herbivore size has been found [25, 26]. Furthermore, the distance that seeds may be dispersed depends positively on herbivore retention time and body size [24, 25]. However, other factors such as diet quality and seed characteristics (size, hardness and shape) may also affect germination [14, 27].

Seed size and seed mass are important traits in the seedling establishment of a plant species [28]. The number of seeds a plant can produce is related to the mass of the seed produced [29]. The greater seed output of small-seeded species such as *Dichrostachys cinerea* may reach a greater proportion of potential establishment sites than large-seeded species e.g. *Acacia nilotica* [30]. The lower output of large-seeded species may be compensated for during seedling establishment, as seedlings from large seeds are generally better at tolerating stresses such as drought, defoliation, shade and plant competition [31]. Large-seeded species would be expected to show greater seedling recruitment (this is the time when the reserves are deployed from the cotyledons) than small-seeded species [18, 32].

Herbivores have a considerable impact on savanna structure because they trample, urinate and defaecate [33]. Furthermore, intense grazing and trampling may result in low plant cover, low or high soil nutrients (depending on patterns of dung deposition), low water infiltration, reduced water availability and high erosion [15, 34], especially on a wet clay soil [15, 35]. Trampling may therefore reduce tree seedling establishment and recruitment due to both the inability of seedling roots to penetrate the soil and high water run-off and reduced water availability for seedlings [36].

Multi-factorial experiments have been emphasized as one of the possible ways to understand the causes and develop management strategies to control woody plant encroachment [1, 3, 16]. Environmental factors such as water stress, light availability and leaf litter cover could constrain the establishment and recruitment of tree seedlings [37]. This may suggest that seeds recovered from herbivores are not necessarily guaranteed to germinate [24, 26]. Additionally, seedling establishment and recruitment, which are pre-requisites for maintaining or increasing tree abundance and are influential in woody plant encroachment, are not assured [3].

Overall, there is unlikely to be a single cause of woody plant encroachment, but rather a combination of interacting factors [2, 15]. To better understand the mechanisms involved, we studied the effects of gut passage (goats and cattle), dung (nutrients), grass competition, fire and trampling on seedling emergence, seedling establishment and recruitment of *Dichrostachys cinerea* and *Acacia nilotica* seeds.

## Materials and Methods

### Study area

**Fig 1:**
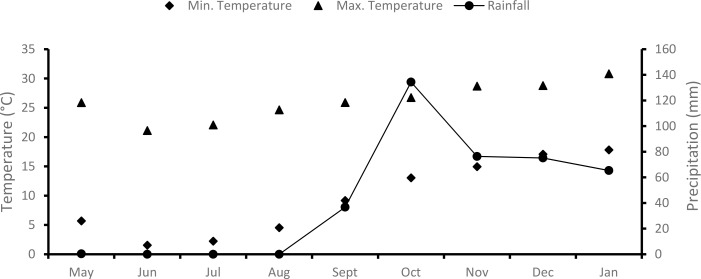
Mean monthly precipitation and minimum and maximum temperatures for 2012–2013 at the Agricultural Research Council’s Roodeplaat Experimental Farm.

The study was done at the Agricultural Research Council‘s Roodeplaat Experimental Farm, Gauteng province, South Africa (28°19´E, 25°35´S). The natural vegetation component of the farm used for livestock production and game makes up an area of approximately 2100 ha. The vegetation type of Roodeplaat is classified as Marikana Thornveld, which is characterized by open *Acacia karroo* and *Acacia caffra* woodlands in the valleys [38]. The main grass species on the site were *Digitaria eriantha* and *Pennisetum clandestinum*. The general soil type is Hutton. The mean annual rainfall is 646 mm, and the minimum and maximum summer and winter temperatures are 20–29°C and 2–16°C, respectively. No watering of seeds was applied throughout the trial; seeds relied only on natural rainfall (Fig. 1).

### Field seedling emergence and seedling establishment

The experimental design consisted of 1 × 1 m plots, with 50 seeds per plot planted at 1 cm depth in the soil [39]. All plots were separated by a 1 m buffer zone. The seedling emergence trial consisted of a completely randomized design with five factors replicated three times per treatment. These factors are: 1) passage through goats or cattle or unpassed/untreated seeds (i.e. not ingested), 2) dung and control (no dung), 3) grass competition and mowed grass, 4) fire and control (no fire), and 5) trampling and control (no trampling) (Table 1).

**Table 1:** The seedling emergence trial consisted of a completely randomized design with three replicates per treatment: passage through goats or cattle or unpassed/untreated seeds, dung, grass competition, fire and trampling.

Gut passage through goats		Gut passage through cattle		Unpassed/untreated seeds (i.e. not ingested by goats or cattle	
Dung	Control (no dung)	Dung	Control (no dung)	Dung	Control (no dung)
Fire	Control (no fire)	Fire	Control (no fire)	Fire	Control (no fire)
Grass competition	Mowed grass	Grass competition	Mowed grass	Grass competition	Mowed grass
Trampling	Control (no trampling)	Trampling	Control (no trampling)	Trampling	Control (no trampling)

Fifty *D*. *cinerea* seeds and 50 *A*. *nilotica* seeds retrieved from goats and unpassed/untreated seeds were planted per plot (50 *D*. *cinerea*, 50 *A*. *nilotica* and 50 unpassed/untreated seeds of each species were planted in separate plots) in three replicates with 16 combinations (96 plots and 4800 seeds). Another 50 *D*. *cinerea* seeds and 50 *A*. *nilotica* seeds retrieved from cattle and untreated seeds were planted per plot in three replicates with 16 combinations (96 plots and 4800 seeds). A total of 14400 seeds were planted in 192 plots for goats, 192 plots for cattle and 192 plots for controls (untreated seeds by passage through the gut of goats or cattle).

### Goats, cattle and unpassed/untreated seeds and ethics statement

Twenty Bonsmara cows and 20 indigenous female goats were divided into two groups of 10 per animal species. Each animal was kept in pens for the duration of seed feeding and recovery. Each animal was fed ground *Digitaria eriantha* hay and water individually in pens. One cow from each group of 10 cows was fed either 1500 *D*. *cinerea* seeds or *A*. *nilotica* seeds mixed with *D*. *eriantha* hay in the feeding trough. One goat from each group of 10 goats was also fed 1500 *D*. *cinerea* or *A*. *nilotica* seeds mixed with *D*. *eriantha* hay in the feeding trough. All experimental animals were allowed to consume seeds for 24 hours, after which the remaining seeds were collected. Retrieved undamaged seeds following techniques outlined by [14] were used during the field seedling emergence trial. This study was approved by the Animal Production Institute Ethics Committee (APIEC).

### Grass competition

The mowed-grass treatments were cut to ground height before planting using a grass trimmer and thereafter every month for the duration of the experiment to simulate the direct effect of grazing (reduced grass competition) using grass shears. Precautions were taken to conserve tree seedlings when cutting grasses around them.

### Dung fertilization

Dung provides a suitable nutritive medium for seedling establishment [40]. Dung collected from experimental animals (goats and cow) after the seed retrieval (i.e. when there were no seeds in the dung) was used for planting to simulate the effect of dung as nutrient input. About 60 cm^3^ of homogenized fresh dung was applied to each seed in dung plots just after planting [15].

### Trampling

Twenty cattle were corralled for 15 days at the trampling treatment site, prior to planting. A goat trampling treatment was not done because their effects are considered to be negligible [41].

### Fire

Grasses are superior competitors to tree seedlings for resources such as water, light, space and nutrients [42, 43, 44]. The fire treatment was applied using old dry grass bales prior to seed planting to ensure that there was a complete, clean burn across the plot. Two dry bales were evenly spread on fire plots to ensure that all (above-ground) grasses were burnt.

### Monitoring of field seedling emergence trial

Seedling emergence and establishment were monitored over three seasons (winter, spring and summer; i.e. from May 2012 to January 2013). The (dry) winter period is the time during which animals consume and distribute seeds of different woody plant species such as *D*. *cinerea* and *A*. *nilotica* seeds. Monitoring seedling establishment during the subsequent (wet) spring and summer seasons was to capture emergence and establishment during the wet season. Seeds were monitored monthly to record emerged seeds and seedlings that died. Seedling recruitment was determined from the difference between the seeds emerged and seedlings that had died since the beginning of the experiment [15].

### Statistical analysis

The germination experiment was subjected to a (3×2^5^) factorial analysis of variance (ANOVA) with the following main factors: seeds retrieved from two animal species ((goats, cattle) and unpassed/untreated seeds (i.e. not ingested by goats or cattle)), two seed species (*A*. *nilotica*, *D*. *cinerea*), two dung treatment levels (dung and no dung), two fire treatment levels (fire and no fire), two trampling treatment levels (trampling and no trampling) and two grass treatment (grass and mowed grass). The trampling treatment was only applied for cattle because goats caused little trampling damage. We analysed the interaction effects of (seed treatment) seed passage through the gut, seed species, dung fertilization, grass competition, trampling and fire on seed germination, seedling establishment and seedling recruitment after a logit transformation [45]. We tested for normality of residuals and homogeneity of variance. Repeated measurements were included in the analysis as a sub-plot factor [46]. The standardized residuals were tested for normality [47]. Differences between means were considered significant at 5% level using a Fisher’s LSD (Least Significant Difference) *post hoc* test. The analysis was done using SAS statistical software [48] for a completely randomized design.

## Results

### Dry season (winter)

**Fig 2:**
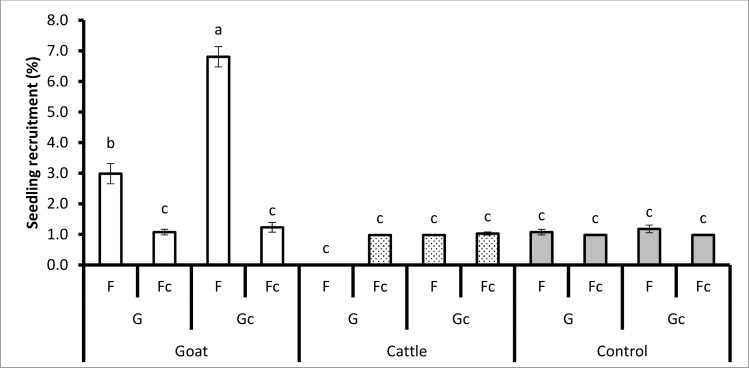
The treatment combination effects of animal species (goats, cattle), unpassed/untreated seeds (i.e. not ingested), grass (G) (and mowed grass (Gc)), fire (F) (and no fire (Fc)) on seedling recruitment during the dry season. Bars represent standard errors (S.E.). Same letters on the bars mean that P > 0.05. A LSD *post hoc* test was used.

The interaction of animal species, grass and fire had a significant effect on seedling emergence (*P* < 0.0167), seedling survival (*P* < 0.0317) and seedling recruitment (*P* < 0.0052, Table 2). Seedling emergence was significantly greater for the treatment combination of seed treatment (passage through the gut of goats), (mowing) mowed grass (Gc), and fire (F) (8.68% ± 0.36) than passage through the gut of goats, grass competition, and fire (4.47% ± 0.24). The treatment combination of seed treatment i.e. passage through the gut of goats, mowed grass (Gc), and fire (F) was greater (6.81% ± 0.33) than passage through the gut of goats, grass competition (G), and fire (F) (2.98% ± 0.33) for seedling recruitment (Fig. 2).

**Table 2:** *F* values and *P* values for the effects of the seven treatments and their interactions and their interactions on seedling emergence, seedling survival and seedling recruitment.

Treatments	Dry Season						Wet Season					
	Germination		Survival		Recruitment		Germination		Survival		Recruitment	
	F	P	F	P	F	P	F	P	F	P	F	P
Animal spp.	61.2	<.0001	51.3	<.0001	60.9	<.0001	14.1	<.0001	8.8	0.0003	11.8	<.0001
Seed spp.	11.0	0.0012	10.4	0.0016	10.3	0.0017						
Fire	41.1	<.0001	40.4	<.0001	48.9	<.0001	29.0	<.0001	28.7	<.0001	25.1	<.0001
Animal spp. × grass × fire	4.2	0.0167	3.6	0.0317	5.5	0.0052						
Animal spp. × dung × grass	4.5	0.0136	3.9	0.0227	3.6	0.0312						
Animal spp. × seed spp. × dung × fire	3.8	0.0236										
Animal spp. × seed spp. × grass × fire	3.7	0.0279										
Seed spp. × dung × grass							6.9	0.0099	4.8	0.0305	8.8	0.0036
Seed spp. × dung × fire			7.0	0.0092								

Dashes (-) = non-significant. Only significant interacting factors are shown. Dashes are used where non-significant main and interacting factors exist (e.g. for *Seed spp*. in the *Wet season*).

**Fig 3:**
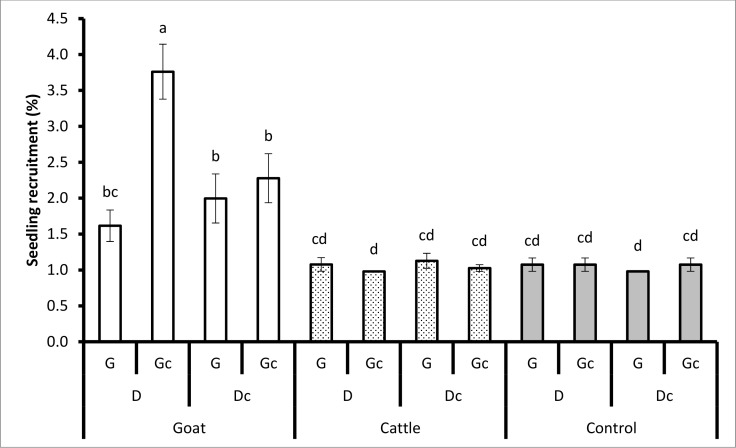
The treatment combination effects of animal species (goats, cattle), unpassed/untreated seeds (i.e. not ingested), dung (D) (and no dung (Dc)), and grass (G) (and mowed grass (Gc)) on seedling recruitment during the dry season. Bars represent standard errors (S.E.). Same letters on the bars mean that P > 0.05. A LSD *post hoc* test was used.

During the dry season, seedling emergence (*P* < 0.0136), seedling survival (*P* < 0.0227) and seedling recruitment (*P* < 0.0312) were significantly affected by animal species, dung and grass (Table 2). Seeds retrieved from goats, planted with dung (D), and mowed grass (Gc) had significantly greater emergence (4.71% ± 0.43) than seeds retrieved from goats, dung (D), and unmowed grass (G) (1.78% ± 0.27). The same was true for seedling survival and seedling recruitment (Fig. 3). Surprisingly, there was no significant difference in seedling emergence, seedling survival and seedling recruitment from seeds retrieved from cattle and unpassed/ untreated seeds, planted with dung (D) or no dung (Dc) and grass (G) or mowed grass (Gc) (Fig. 3).

The interaction effect of animal species, seed species, dung and fire had a significant effect on seedling emergence (*P* < 0.0236; Table 2). The treatment combinations of seed treatment (passage through the gut of goats), *A*. *nilotica* seeds, no dung (Dc), fire (F) (13.96% ± 0.33) and passage through the gut of goats, *A*. *nilotica* seeds, dung (D), and fire (F) (10.53% ± 0.42) emerged significantly more than the treatment combinations of seed treated by goats, *A*. *nilotica* seeds, no dung (Dc), and no fire (Fc) (1.18% ± 0.19) and seed treated by goats, *A*. *nilotica* seeds, dung (D), and no fire (Fc) (1.48% ± 0.42). There was a significant difference in *D*. *cinerea* seedling emergence between the treatment combination of seed treated ingested by goats, with dung (D), and with fire (F) (4.39% ± 0.50) and seed treated by goats, no dung (Dc), and with fire (F) (1.71% ± 0.39).

**Fig 4:**
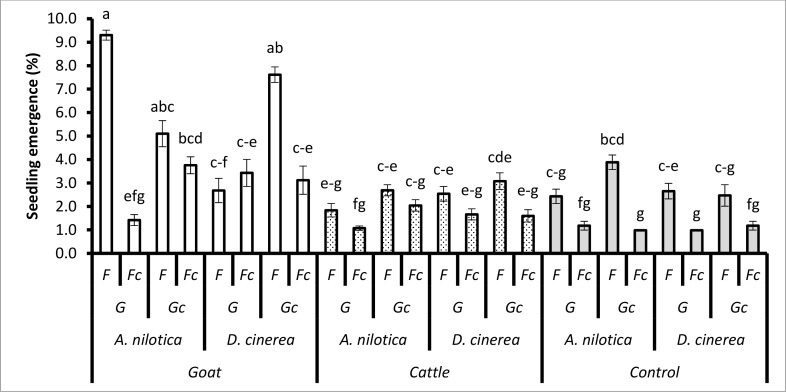
The treatment combination effects of animal species (goats, cattle), unpassed/untreated seeds (i.e. not ingested), seed species (*A*. *nilotica*, *D*. *cinerea*), grass (G) (and mowed grass (Gc)), fire (F) (and no fire (Fc)) on seedling emergence during the dry season. Bars represent standard errors (S.E). Same letters on the bars mean that P > 0.05. A LSD *post hoc* test was used.

There was a significant interaction of animal species, seed species, grass and fire on seedling emergence (*P* < 0.028; Table 2). During the dry season, *A*. *nilotica* seedling emergence was significantly higher in seeds treated by goats, with the treatment combination of grass (G), and with fire (F) (9.30% ± 0.21) than seed treated by goats, with grass (G), and no fire (Fc) (1.42% ± 0.24). During the dry season, *D*. *cinerea* seedling emergence was significantly higher in the treatment combination of seeds treated by goats, with mowed grass (Gc), and fire (F) (7.62% ± 0.33) than seeds treated by goats, with mowed grass (Gc), and no fire (Fc) (3.12% ± 0.60; Fig. 4). Emergence of unpassed/untreated *A*. *nilotica* seedlings with the treatment combination of mowed grass (Gc) and fire (F) (3.88% ± 0.31) was significantly greater than unpassed with mowed grass (Gc), and no fire (Fc) (0.98% ± 0.00; Fig. 4).

**Fig 5:**
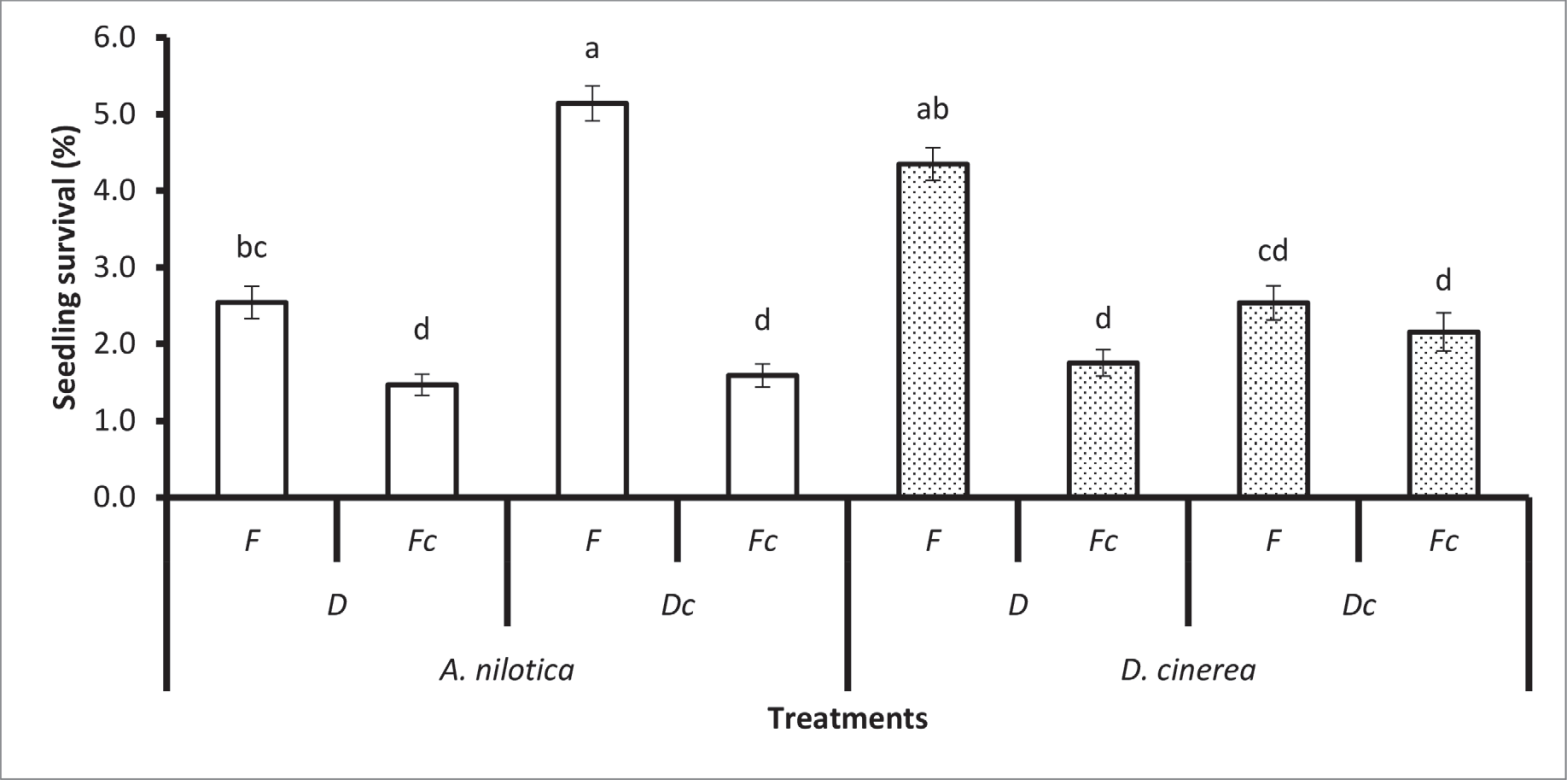
The treatment combination effects of seed species (*A*. *nilotica*, *D*. *cinerea*), dung (D) (and no dung (Dc)), fire (F) (and no fire (Fc)) on seedling survival during the dry season. Bars represent standard errors (S.E). Same letters on the bars mean that P > 0.05. A LSD *post hoc* test was used.

The interaction of seed species, dung and fire had a significant effect on seedling survival (*P* < 0.0092; Table 2). The treatment combination of *Acacia nilotica* seeds, no dung (Dc), fire (F) (5.14% ± 0.23) and *D*. *cinerea* seeds, dung (D), fire (F) (4.35% ± 0.21) survived significantly better than *A*. *nilotica* seeds, no dung (Dc), no fire (Fc) (1.59% ± 0.15) and *D*. *cinerea* seeds, dung (D), no fire (Fc) (1.75% ± 0.17; Fig. 5).

Seeds retrieved from goats (3.59% ± 0.16) recruited significantly better than seeds retrieved from cattle (1.93% ± 0.09) and control/untreated seeds (1.69% ± 0.11). Significantly more *A*. *nilotica* seeds (1.73% ± 0.07) recruited than *D*. *cinerea* seeds (1.62% ± 0.06). The fire (2.95 ± 0.11) treatment (which was used to remove the above-ground grass) had significantly more seedling recruitment than no fire (1.61 ± 0.09).

### Wet season (summer)

**Fig 6:**
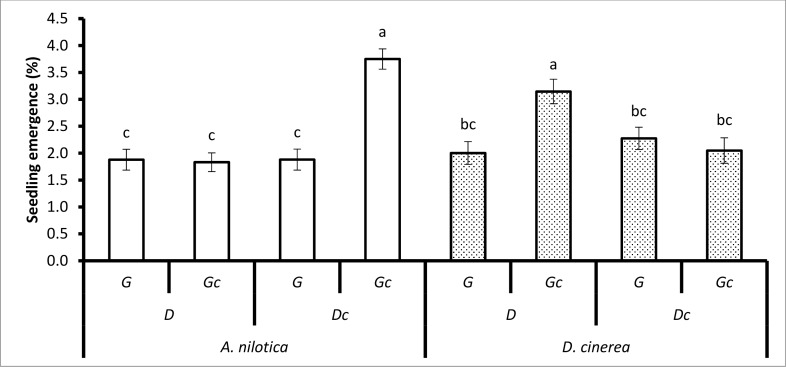
The treatment combination effects of seed species (*A*. *nilotica*, *D*. *cinerea*), dung (D) (and no dung (Dc)), grass (G) (and mowed grass (Gc)) on seedling emergence during the wet season. Bars represent standard errors (S.E). Same letters on the bars mean that P > 0.05. A LSD *post hoc* test was used.

The interaction of seed species, dung and grass had a significant effect on seedling emergence (*P* < 0.0099), seedling establishment (*P* < 0.0305) and seedling recruitment (*P* < 0.0036; Table 2). The seedling emergence of *A*. *nilotica* with the treatment combination of no dung (Dc) and mowed grass (Gc) (3.75% ± 0.19) was significantly greater than with no dung (Dc) and grass (G) (1.88% ± 0.17). Seedling emergence of *D*. *cinerea* with the treatment combination of dung (D) and mowed grass (Gc) (3.15% ± 0.23) was significantly greater than *D*. *cinerea* with dung (D) and grass (G) (2.00% ± 0.21). Seedling emergence of *A*. *nilotica* with the treatment combination of no dung (Dc) and mowed grass (Gc) (3.75% ± 0.19) was significantly greater than *A*. *nilotica* with no dung (Dc) and unmowed grass (D) (1.88% ± 0.18) (Fig. 6).

## Discussion

### Seedling emergence, seedling survival and recruitment

The results of this study showed that goat-ingested seeds (3.94%) had significantly greater germination (3.94%) than cattle-ingested seeds (1.97%) and control/untreated seeds (1.74%) (i.e. no passage through the gut of livestock). Previous author [25] has shown that seed germination increases with animal body size. The opposite results were found in this study. However, these results are consistent with those of [15] in KwaZulu-Natal, South Africa. Even though cattle and untreated seeds were not significantly different in terms of seedling emergence and seedling recruitment, it is clear that acid scarification in the gut of herbivores usually favours seedling emergence [14, 15]. Longer retention of seeds ingested by large bodied-animals, e.g. cattle, may result in substantial damage to the seeds [23], which may cause significantly lower seedling emergence after cattle ingestion than from goats. Recovery of viable seeds depends on the interaction of different factors such as seed characteristics (hardness, shape), associated diet and animal species, which may result in increased or decreased germination [49]. For instance, in this study, there were relatively more damaged seeds observed from cattle ingestion than goat ingestion [49]. The large *A*. *nilotica* seeds recruited significantly better than the smaller *D*. *cinerea* seeds. These results are consistent with those of [31] who reported seedlings from large-seeded species are expected to show a greater seedling recruitment than small-seeded species due to the reserves deployed in the cotyledons.

Seedling emergence and seedling survival increased significantly with fire, grass mowing and seed passage through the gut of goats but not with dung fertilization. Conversely, dung addition increased seedling recruitment in the goat with grass treatment. The results in this study suggest that dung may provide a suitable nutritive medium for seedling emergence, establishment and recruitment [40, 50], contrary to the results reported in [15]. The non-significant seedling emergence and seedling survival with addition of dung may have been caused by dry dung and creation of a hard dung layer [15, 51]. In addition, dung may not have been decomposed and nutrients incorporated into the soil [52], which resulted in a negative effect of dung on seedling emergence. Another possible reason might be competition with other plant species [53].

The most interesting results were the positive effect of fire treatments on seedling emergence and seedling recruitment in this study. Grasses are superior competitors to tree seedlings for resources such as water, light, space and nutrients [54, 55, 56]. For this reason, *A*. *nilotica* and *D*. *cinerea* seedlings and other woody plant species may be disadvantaged by germinating and surviving among grasses without fire [57]. The use of fire to remove the above-ground grass had the most important effect on seedling emergence and seedling recruitment, most probably because of less dense grass cover and therefore reduced competition between tree seedlings and grasses [58, 59]. In addition, the possibility of ash as a fertilizer may have played a role but because this factor was not considered; therefore, no conclusive answers can be given. It is clear from this study that combinations of interacting factors (e.g. animal species, seed species, grass competition, dung fertilization and fire) influence seedling recruitment of *D*. *cinerea* and *A*. *nilotica* seeds. Regardless of whether there was fire or not, dung or no dung, the effect of livestock (especially goats) played an important role in seed scarification. Furthermore, livestock may also disperse viable seeds away from the mother tree, which may favour competition with adult trees [60].

Seedling emergence and survival of seedlings throughout the dry season (when water is scarce) plays a crucial role in plant population dynamics. Rainfall plays an important role in germination, seedling establishment and recruitment [3, 58, 61]. The effect of fire treatment, which removed grass competition, positively affected survival and recruitment of *D*. *cinerea* and *A*. *nilotica* seedlings [15, 54, 56]. In addition, soil moisture from precipitation [3, 62] is the most limiting factor in the growth of tree and grass seedlings. Grasses are able to take up and use rainwater at a much faster rate than woody trees [63, 64]. Less or insufficient soil moisture will reach the deeper subsurface layers of the soil where the roots of woody plants dominate, thus putting the trees at a disadvantage [65]. The disturbance created by the selective removal of grass cover by heavy grazing allows the infiltration and percolation of water to deeper soil layers exploited by the roots of woody vegetation, conferring a competitive advantage to trees [65, 66]. As a result, woody vegetation may then recruit *en masse* in the patches opened up by grazing disturbances [1].

### Management applications

We showed that seed ingestion by goats improves seedling emergence. In addition, the fire treatment (used to remove the above-ground grasses) and mowed-grass treatment (used to simulate the indirect effect of grazing) played an important role by reducing above-ground grass competition with tree seedlings for resources. Regardless of the relatively low seedling emergence, seedling survival and recruitment, this study shows that the direct and indirect effects of gut passage, grass competition and precipitation were important in the recruitment of *D*. *cinerea* and *A*. *nilotica* seedlings, and may consequently contribute to woody plant encroachment. Overall, we showed in this experiment that the passage of seeds through the guts of different animals improves germination. However, improved germination may not directly lead to woody plant encroachment. It was clear that interactions of various other factors (such as fire and simulated grazing) both directly and indirectly may lead to woody plant encroachment.
